# The Phosphatase CSW Controls Life Span by Insulin Signaling and Metabolism Throughout Adult Life in *Drosophila*

**DOI:** 10.3389/fgene.2020.00364

**Published:** 2020-05-07

**Authors:** Leonardo R. Ruzzi, Pablo E. Schilman, Alvaro San Martin, Sergio E. Lew, Bruce D. Gelb, Mario R. Pagani

**Affiliations:** ^1^Department of Physiology and Biophysics, School of Medicine, National Scientific and Technical Research Council, University of Buenos Aires, Buenos Aires, Argentina; ^2^Department of Biodiversity and Experimental Biology, Faculty of Exact and Natural Sciences, National Scientific and Technical Research Council, University of Buenos Aires, Buenos Aires, Argentina; ^3^Institute of Biomedical Engineering, Faculty of Engineering, University of Buenos Aires, Buenos Aires, Argentina; ^4^Mindich Child Health and Development Institute, Icahn School of Medicine at Mount Sinai, New York, NY, United States; ^5^Department of Pediatrics, Icahn School of Medicine at Mount Sinai, New York, NY, United States; ^6^Department of Genetics and Genomic Sciences, Icahn School of Medicine at Mount Sinai, New York, NY, United States

**Keywords:** CSW loss and gain-of-function mutations, life span, *Drosophila*, insulin signaling, metabolism

## Abstract

Noonan syndrome and related disorders are caused by mutations in genes encoding for proteins of the RAS-ERK1/2 signaling pathway, which affect development by enhanced ERK1/2 activity. However, the mutations’ effects throughout adult life are unclear. In this study, we identify that the protein most commonly affected in Noonan syndrome, the phosphatase SHP2, known in *Drosophila* as *corkscrew* (CSW), controls life span, triglyceride levels, and metabolism without affecting ERK signaling pathway. We found that CSW loss-of-function mutations extended life span by interacting with components of the insulin signaling pathway and impairing AKT activity in adult flies. By expressing *csw-RNAi* in different organs, we determined that CSW extended life span by acting in organs that regulate energy availability, including gut, fat body and neurons. In contrast to that in control animals, loss of CSW leads to reduced homeostasis in metabolic rate during activity. Clinically relevant gain-of-function *csw* allele reduced life span, when expressed in fat body, but not in other tissues. However, overexpression of a wild-type allele did not affect life span, showing a specific effect of the gain-of-function allele independently of a gene dosage effect. We concluded that CSW normally regulates life span and that mutations in SHP2 are expected to have critical effects throughout life by insulin-dependent mechanisms in addition to the well-known RAS-ERK1/2-dependent developmental alterations.

## Introduction

Normal tissue functions, including growth, maintenance, and regeneration, depend on the availability of energy provided by food or reservoirs like glycogen and lipids. In mammals, growth depends on growth hormone and, in turn, insulin-like growth factors (e.g., IGF1), which promote the activation of the RAS-ERK1/2 signaling pathway. On the other hand, insulin signaling promoting the activation of PI3K-AKT signaling pathway plays an essential role in growth and energy balance. In *Drosophila*, both functions are mediated by the insulin and insulin-like growth factor signaling (IIS) pathway downstream of a single insulin receptor (InR) (Reyes-DelaTorre et al., 2012; Rajan and Perrimon, 2013). Like humans, *Drosophila* regulates its levels of circulating carbohydrates and stores excesses as glycogen and lipids, mainly triglycerides (TGs), in the fat body (Teleman, 2010). A large number of studies have shown evolutionarily conserved functional signaling mechanisms between fruit flies and vertebrates, like the insulin/IGF/target of rapamycin pathway (Oldham and Hafen, 2003; Reyes-DelaTorre et al., 2012). Accumulating evidence shows that insulin signaling affects metabolism in vertebrates and invertebrates with profound effects on survival (Kenyon et al., 1993; Böhni et al., 1999; Clancy et al., 2001; Tatar et al., 2001; Blüher et al., 2003).

The protein tyrosine phosphatase (PTP) SHP2, encoded by *PTPN11* (OMIM# 176876), is a ubiquitously expressed protein acting downstream of many receptor tyrosine kinases, including InRs (Kuhné et al., 1993; Perkins et al., 1996). SHP2 is involved in development and developmental disorders such as Noonan syndrome (NS; OMIM# 163950) and NS with multiple lentigines (NSML; OMIM# 151100; formerly, LEOPARD syndrome) (Tartaglia et al., 2001; Digilio et al., 2002). These disorders are part of a larger group of disorders named the RASopathies, caused largely by gain-of-function (GOF) mutations that enhance the activity of the RAS-ERK1/2 signaling pathway (Tartaglia and Gelb, 2005; San Martín and Pagani, 2014; Jindal et al., 2015). SHP2 is a proto-oncogene, and growth alterations as well as higher incidence of malignancies have been documented in the RASopathies (Tartaglia et al., 2003; Tartaglia and Gelb, 2005; Chan and Feng, 2007). Moreover, SHP2 is involved in proliferation and differentiation of stem cells in different tissues and developmental stages (Qu and Feng, 1998; Ke et al., 2007; Wu et al., 2009; He et al., 2013). Of note, normal stem cell –functions are required for tissue homeostasis and regeneration throughout life, for which insulin signaling seems to be critical in a nutrition-dependent manner (Biteau et al., 2010; Shim et al., 2013).

Whereas mutant mice lacking Shp2 die during development, mice heterozygous for the knock-out allele showed no effect on insulin sensibility or insulin signaling activity (Arrandale et al., 1996; Saxton et al., 1997). The main effect of a reduction of SHP2’s phosphatase activity in all cells of an animal remained elusive until recently. Reduced SHP2 function by a loss-of-function (LOF) mutation T468M modeling NSML showed increased tolerance to both insulin and high-fat diets (Tajan et al., 2014). However, this and other NSML-associated mutations (i.e., T468M and Y279C) showed reduced phosphatase activity, but GOF effects in development as well as enhanced ERK1/2 signaling activity during development or after insulin injection (Oishi et al., 2009; Tajan et al., 2014) presumably through phosphatase-independent SHP2 functions (Stewart et al., 2010). Of note, NSML and NS are also caused by GOF mutations in RAF1 (Pandit et al., 2007).

Taken together, this evidence suggested that mutations in SHP2 might produce an altered regulation of mechanisms involved in metabolism and tissue homeostasis. However, the effect of a global SHP2 reduction on metabolism and life span, as well as the pathological implications, remains elusive. Here, we examined the effect of genetic manipulations of SHP2 on life span and metabolism using a *Drosophila* model system.

## Materials and Methods

### *Drosophila* Stock and Culture

Flies were raised on standard yeast and sucrose-based food, at 25°C or 29°C and 65% humidity, on a 12-h light/dark cycle, unless otherwise indicated. The control line *w*^1118^ was generated by crossing separate inbred *w*^1118^ lines (FBst0003605) and established before use. This ensured a healthy life span by avoiding inbreeding depression in the control group. The following stocks were obtained from the Bloomington *Drosophila* Stock Center: *w*^1118^, *csw^lf^/FM7* (FBst0023873), *csw^6^/FM7* (FBst0023876), *FB-r4Gal4* (FBst0033832), *Arm-Gal4* (FBst0001560), *elav^C155^-Gal4* (FBst0000458), *DJ757-Gal4* (FBst0008184), *Ilp2-Gal4* (FBst0037516), *esg-Gal4^5730^* (FBst0026816), *chico^1^/cyo* (FBst0010738), *InR^E19^/TM2* (FBst0009646), and *Bx^MS1096^-Gal4* (FBst0008860) and from the Vienna *Drosophila* RNAi Center *UAS-csw-RNAi* (FBst0454218). The following lines were kindly provided by B. Gelb (*UAS-csw^WT^* and *UAS-csw^T73I^*) and Y. Zhong (*Cy/Sp;Sb/Ser*). Mutants and transgenic flies were backcrossed to the control line *w*^1118^ for five generations to eliminate differences in genetic background. These lines are described in flybase.org.

### Life Span Analysis in Poor and Rich Diets and Starvation

Female flies were collected within 48 h of eclosion under brief CO_2_ anesthesia (less than 2 min) and kept at a density of 25–30 female flies per vial in controlled 12-h light/dark cycle. For life span experiments, flies were kept at 29°C in 65% humidity, and in some cases, the same experiment was also performed at 25°C. At least three independent experiments were performed for each condition and genotype. Vials were changed three times a week, and the number of dead flies was counted. For life span in poor and rich diets, flies were treated in the same way as life span analysis (29°C in 65% humidity) but collected and kept in vials containing 0.25% or 15% yeast media for poor and rich diets, respectively. For starvation-resistance experiments, flies were raised and collected in the same way as life span experiments and kept at 29°C in controlled 12-h light/dark cycle in vials containing agar 1%. Vials were changed every 3 h seven times a day. Prism (GraphPad) software was used to build survival curves as Kaplan–Meier survival plot and comparisons by log-rank and Gehan–Wilcoxon tests corrected for multiple comparisons.

### Western Blot Analysis

Four adult flies of the corresponding feeding condition were lysed in 50 μl of EBR buffer [2 mM of Cl_2_Ca, 129 mM of NaCl, 4 mM of KCl, and 35 mM of Tris (pH 6.85)] supplemented with phosphatase/protease inhibitor cocktail (Sigma #P2714), NaF (50 mM), and sodium orthovanadate (1 mM). A 12 mg protein/lane and a 24 mg protein/lane were used for pERK and pAKT immunoblots, respectively. Tissue samples were run on 12% sodium dodecyl sulfate–polyacrylamide gel electrophoresis (SDS–PAGE) and transferred to polyvinylidene difluoride (PVDF) (Thermo Scientific). Primary antibodies used were as follows: anti-pAKT^Ser^ ^505^ 1:1,000 (Cell Signaling #4054), anti-AKT 1:1,000 (Cell Signaling #9272), anti-pERK1/2 1:1,000 (Sigma #M8159), and anti-ERK1/2 1:15,000 (Sigma #M5670). Horseradish peroxidase (HRP)-conjugated secondary antibody goat anti-mouse 1:1,000 (Sigma #A5278), goat anti-rabbit 1:5,000 (Sigma #A0545), and Super Signal West Pico (Thermo Scientific) were used for signal detection in an ImageQuant RT ECL (GE Healthcare Life Sciences) device. Activation was quantified by ImageJ 1.47v as phospho-protein normalized to non-phospho-protein (pERK/ERK and pAKT/AKT).

### Immunohistochemistry

For staining against phospho-AKT^Ser^ ^505^, female flies were fasted for 16 h in agar 1% and after that fed in standard fly media for 4 h when animals were anesthetized in iced water. Guts were dissected on ice, immediately fixed in paraformaldehyde (PFA) 4% for 20 min at room temperature, washed with phosphate-buffered saline (PBS) 0.1 M, and incubated with methanol during 5 min at −20°C. Then, guts were rinsed on PBS 0.1 M and incubated with Tris-HCl 150 mM, pH 9.0, at 70°C during 15 min for antigenic exposure. After being rinsed with PBS 0.1 M and incubated with PBS-T during 15 min, guts were blocked with PBS-T and bovine serum albumin (BSA) 2% for 30 min at room temperature and incubated with the primary antibody overnight at 4°C. After that, guts were rinsed on PBS-T and incubated with the corresponding secondary antibody for 2 h at room temperature. For staining against phospho-ERK, female flies were fed for 16 h with H_2_O_2_ 1% in sucrose 5% and, after that, fed with standard fly media for 24 h when animals were anesthetized in iced water processed for immunostaining as before without antigenic exposure. The following primary antibodies were used: rabbit anti-pAKT^Ser^ ^505^ 1:1,000 (Cell Signaling #4054) and mouse anti-pERK1/2 1:1,000 (Sigma #M8159). Secondary antibodies were goat anti-rabbit Alexa Fluor 555 (#A-21429) and goat anti-mouse Alexa Fluor 488 (#A-11029) from Life Technologies. DAPI and propidium iodide were used to stain DNA. Images were collected with BX53 epifluorescence microscope and a Q-color 5 digital camera (Olympus Optical) and processed using Image J 1.47v.

### Triglyceride Determinations

Females flies aged 3–5 days of each genotype were placed in groups of 25–30 in vials containing standard fly media. For TG determinations in hemolymph, flies were cold anesthetized, and hemolymph was extracted by puncturing the thorax with 0.27-mm entomological needle follow by centrifugation at 2,600 *g* for 5 min at 4°C. For TG determinations in abdominal segments, five flies were cold anesthetized, and their abdomen was separated and homogenized in 40 μl of lysis buffer (0.2% Tween 20 and PBS 0.1 M). The TG levels were determined by spectrometry according to manufacturer’s instructions (TG Color GPO/PAP AA Wiener Lab). Each experiment was repeated at least three times.

### Respirometry

A Sable Systems International (SSI) system was used for flow-through respirometry supplemented by an optical activity detector SSI AD-2. Temperature was controlled to 25 ± 0.1°C by a SSI Pelt-5 temperature controller coupled to a SSI Peltier Plate. Room air was pushed through a Drierita–Ascarita–Drierita column to scrub off water vapor and CO_2_ at a standard temperature and pressure (STP)-corrected flow rate of 50 ml min^–1^ by a Side-Trak Model 830 mass-flow valve (Sierra Instrument, CA, United States) connected to a MFC-2 mass-flow controller (SSI). The prepared air is then entered into SSI thermal respirometry chamber (internal volume < 1 ml). Finally, air left the respirometry chamber (having gathered CO_2_ from the insects on its way) and entered the Li-Cor (LI-6251) CO_2_ infrared analyzer (Lincoln, NE, United States; resolution 0.1 ppm of CO_2_). The analog outputs from the analyzers measuring CO_2_, fly’s activity, temperature of the chamber, and air flow rate were connected to a A/D converter (SSI UI-2, 16-bit basic accuracy = 0.05%) and stored in a computer by ExpeData data acquisition software (SSI) for further analyses (Schilman et al., 2011).

An individual female *Drosophila* was transferred from the breeding container to a 2-ml vial and then placed into the chamber. The fly was minimally handled, no thermal or chemical tranquilization was applied, and the experimental temperature was the same to that of the rearing one. The recording began with a baseline segment to establish the zero points for the carbon dioxide and activity analyzers. After that, the CO_2_ released by the fly was measured for 20 to 50 min, plus another baseline. Data points were taken at 1-s intervals (1 Hz).

For each recording, CO_2_ baselines were subtracted assuming a linear drift and converted from parts per million to microliter per hour and to energy units of microwatt using a respiratory quotient (RQ) = 1. Mass-independent metabolic rate was calculated by dividing catabolic flux rates in microwatt by live mass in milligrams raised to the 0.856th power, which is the inter-specific mass scaling exponent for tracheate arthropods (Lighton et al., 2001). Fresh body weight was determined by weighting female flies, under *ad libitum* conditions, in groups of 20–25 flies in a Sartorius 2474 scale.

In order to compare activity from different flies and recordings, an index of activity was calculated. To do this, activity was converted to the absolute difference sum (ADS), that is, the cumulative sum of the absolute difference between all of adjacent data points, data stored into a new different channel, and the index of activity calculated based on the equation: Activity index = ADS range/*N*^∗^60, where ADS range is the difference between maximum and minimum values of ADS activity and *N* is the number of samples or seconds of the recording.

### Climbing Assay

Climbing assays were performed as previously described (Gargano et al., 2005). In brief, 20–30 flies were placed in empty vials and, after 1 min of rest, gently tapped three times to move flies to the bottom. After 4 s, the position of the flies in each tube was registered with a digital camera connected to a PC. The camera was controlled by a MATLAB script that detected the vials’ movement considering as time 0 when the vials stop moving. After 1 min of rest, this procedure was repeated four more times, and the average position of the flies in the vial was calculated, with other MATLAB script, to generate a single data (*N* = 1).

### Statistical Analysis

Statistical analysis was performed using Prism GraphPad 6.01 software. Comparisons of survival curves as Kaplan–Meier survival plot were performed by log-rank and Gehan–Wilcoxon tests corrected for multiple comparisons. Shapiro–Wilk’s test was used to evaluate data normality. For the purpose of checking statistical significance between two groups, unpaired *t*-test or Mann–Whitney test were used for parametric or non-parametric analysis, respectively. One-way ANOVA or Kuskal–Wallis was used for comparison of statistical significance between more than two groups for parametric or non-parametric analysis, respectively. Two-way ANOVA was used for comparison between groups with two factors followed by Sidak’s multiple comparison test. Statistical significance was defined as *p* < 0.05.

## Results

Previous studies in mammalian models found that knock-in LOF or knock-out of SHP2 in specific tissues increased tolerance to insulin (Matsuo et al., 2010; Nagata et al., 2012; Tajan et al., 2014). Because we wanted to determine whether we could use fruit flies to investigate the role of SHP2 in metabolism and life span, we began by examining the effect of reducing CSW in fruit flies on life span.

### Loss-of-Function *csw* Increased Median Life Span and Longevity

**FIGURE 1:**
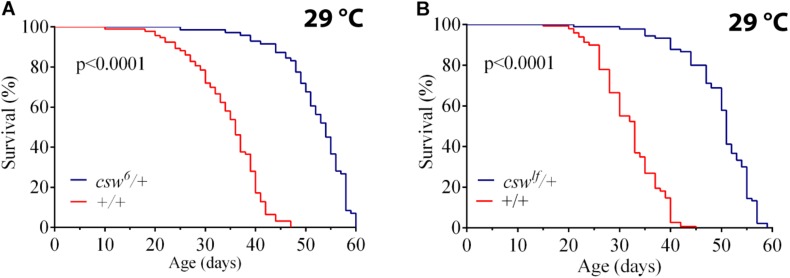
Loss-of-function (LOF) *csw* mutations extend life span. Effect of reducing the tyrosine phosphatase CSW on life span at 29°C. **(A,B)** Life span was extended by the classical *csw* LOF mutations *csw*^6^/+ **(A)** and *csw*^lf^/+ **(B)** compared with a control group (+/+). All the three genotypes share the same genetic background of *w*^1118^. Log-rank test. The number of animals was *n* = 103 to 149, at least from four independent experiments.

To understand the role of SHP2 in survival, we investigated the effect of suppressing CSW function, the orthologous phosphatase in *Drosophila.* First, we ensured that the genotype to be used as control, *w*^1118^, showed a healthy life span similar to that of a wild-type (WT) group (see *Methods*). Then, we analyzed the effect of the weakest hypomorphic allele *csw*^6^ on survival (Perkins et al., 1996), because *csw-RNAi* expressed in the whole body was lethal. For this experiment, we used female fruit flies heterozygous for *csw*^6^, because it is lethal in males (Perkins et al., 1996). The reduced function of CSW in this hypomorphic genotype showed an increase in median life span and maximum life span compared with those in the control group at 29°C (Figure 1A) or at 25°C (Supplementary Figure S1A). This increase in survival showed a similar proportion at both temperatures (Supplementary Figures S1B,C). To confirm that reducing CSW function increased survival, we examined the effect of a second weak hypomorphic allele *csw*^lf^, also using female flies due to male lethality. This second *csw* allele produced a comparable increase in both features of survival (Figure 1B).

We confirmed the LOF effect of *csw*^lf^, which consists of a typical LOF wing vein phenotype (Oishi et al., 2006) (Supplementary Figures S1D,E). Taken together, these observations suggested that LOF *csw* allele in the whole organism increased survival. Next, we focused on potential mechanisms mediating these effects.

### Loss-of-Function *csw* Extended Life Span by Interacting With Insulin/Insulin-Like Growth Factor Signaling and Reducing AKT Activation

**FIGURE 2:**
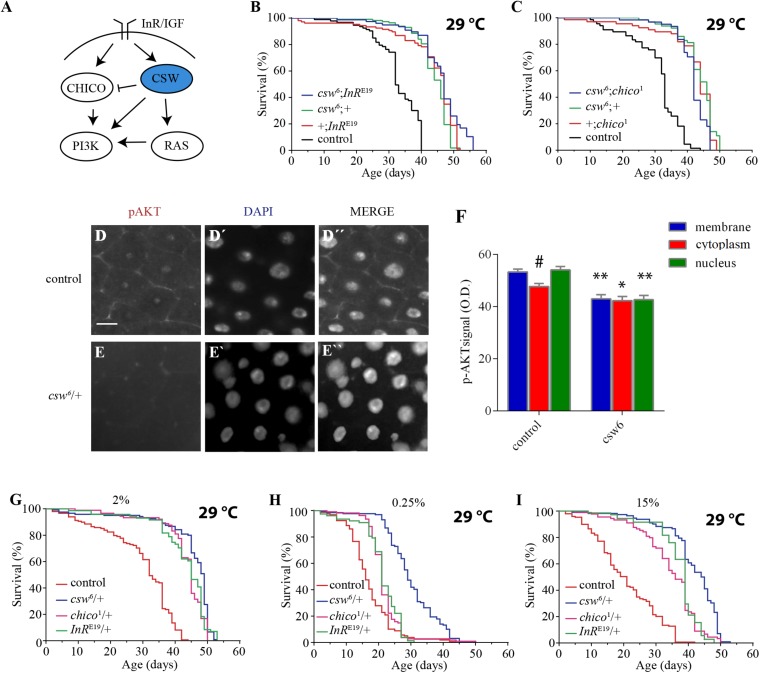
Loss-of-function (LOF) *csw* interacts with the insulin pathway reducing AKT activation and extended life span in poor and rich diets. **(A)** Simplified schematic representation of the roles of the vertebrate tyrosine phosphatase SHP2 and ortholog of CSW in fruit flies. Arrows indicate positive regulatory effects, and blunt arrow indicates negative regulatory effect. Note the predominant positive effect of SHP2. **(B,C)** Non-additive effect on life span curves by genetic interaction of LOF *csw*^6^ with classical insulin mutants, *InR^E19^* and *chico*^1^. **(B)** Double mutant *csw*^6^; *InR^E19^* compared with single mutants (*csw*^6^;+ and +;*InR^E19^*) and +;+ (control). **(C)** Double mutant *csw*^6^; *chico*^1^ compared with single mutants (*csw*^6^;+ and +;*chico*^1^) and +;+ (control). **(D–F)** Immunostaining showing reduced AKT activation in LOF *csw* posterior midguts, using anti-pAKT. Representative staining in control **(D)** and LOF *csw*^6^ gut **(E)** showing pAKT, DNA staining **(D’,E’)**, and merged staining **(D”,E”)**. Scale bar 10 μm. **(F)** Average optical density of pAKT, corresponding to plasma membrane, cytoplasm, and nucleus for mutant (*csw^6^/*+) and control (+*/*+) fruit flies. Each bar represents 40 data points from four different animals. Asterisks indicate significant reduction in *csw^6^/*+ compared with control guts, ^∗∗^*p* < 0.0001; ^∗^*p* < 0.05; and # indicates significant reduction in cytoplasm compared with plasma membrane or nucleus in control group, two-way ANOVA, Sidak’s multiple comparison test. **(G–I)** Survival curves showing extended life span in poor and rich yeast diets of *csw*^6^/+ and classical insulin mutants, *InR^E19^*/+ and *chico*^1^/+, compared with control group +/+ cultured in fly media containing the concentrations of yeast: 2% **(G)**, 0.25% **(H)**, and 15% **(I)**. The three mutants showed extended life span as compared with control group, *p* < 0.0001, log-rank test, corrected for multiple comparisons. For the panels **(B,C,G–I)**, the number of animals was *n* = 61 to 129, at least from three independent experiments.

Reduction in the IIS pathway has been shown to increase life span in organisms ranging from yeast to mammals. Consistent with this, SHP2 has been reported to be a positive regulator of both insulin-dependent activation of ERK1/2 through RAS and IGF1-dependent activation of PI3K in mammalian cell cultures (Figure 2A) (Yamauchi et al., 1995; Wu et al., 2001). In addition, SHP2 also participates as a negative regulator of the insulin-dependent activation of PI3K through the InR substrate (IRS), the mammalian ortholog of *chico* (Myers et al., 1998) (Figure 2A). Therefore, we hypothesized that the life span extension observed in *csw*^6^ flies might be due to a decline in CSW as a positive regulator on the IIS pathway.

To begin examining this possibility, we performed gene interaction studies using LOF alleles of genes with well-established effect on life span, *InR* and *chico*. If the effect of *csw*^6^ on survival is mediated by a mechanism independent of the IIS pathway, an additive effect would be expected in an *InR* and *csw* double mutant. However, if *csw*^6^ extended life span through the IIS pathway, an interaction should be observed, as previously reported for *InR* and *chico* (Böhni et al., 1999). To test this, we analyzed the effect of a weak LOF allele, *InR^E19^*, which has been shown to reduce insulin-like receptor activity in heterozygous fruit flies (Tatar et al., 2001). For these experiments, we used female fruit flies to make it comparable with LOF *csw* females. As expected, the *InR^E19^* allele increased survival compared with the control group +/+ and was similar to *csw^6^/*+. The double mutant (*csw^6^/*+; *InR^E19^/*+) did not show an additive effect in life span (Figure 2B).

To further support our observation that *csw*^6^ extended life span by interacting with molecular components of the IIS signaling, we examined the interaction of *csw*^6^ with *chico*^1^, which has been shown to extend life span (Clancy et al., 2001). Similar to what we observed for *InR^E19^*, *chico^1^/*+ increased survival, whereas the double mutant (*csw^6^/*+; *chico^1^/*+) did not show an additive effect (Figure 2C). Together, these results suggested that LOF *csw* might be extending life span through IIS reduction.

The genetic interaction data suggested that LOF *csw* reduces the insulin signaling pathway, resulting in life span extension. To confirm this interpretation, we examined the activity levels of AKT and ERK as a read-out of the activity levels of the IIS pathway by western blot in the whole body of LOF *csw* flies fed *ad libitum*, starved, and at different times after refeeding. The response of AKT and ERK activation to the feeding conditions in the LOF *csw* was undistinguishable from that of the control group (Supplementary Figures S2A–D). We reasoned that possible differences in specific tissues might be diluted in the mix of tissues of the whole body. To examined this possibility, we used immunostaining to assess AKT and ERK activation in the gut, an important organ in determining survival in an IIS-dependent manner (Biteau et al., 2011; Rera et al., 2012).

We examined adult LOF *csw* and control flies after 16 h of fasting followed by 4 h of refeeding. We found reduced pAKT staining in the CSW mutant compared with control in areas corresponding to the plasma membrane, cytoplasm, and nucleus (Figures 2D–F). In addition, in the control group, there was a higher activation of AKT in the plasma membrane or in the nucleus compared with the cytoplasm (*p* < 0.05), whereas there was no such difference in the CSW mutant. Moreover, no staining for the activated form of ERK was detected. We confirmed that ERK was activated in the gut in our flies after tissue damage as has been previously reported (Jiang et al., 2011) (Supplementary Figure S3), validating that assay.

These results showed that LOF *csw* interacts with IIS pathway components and impairs AKT activation and localization in specific tissues with no effect on ERK activity in adult animals. Therefore, we concluded that CSW normally participates in the IIS pathway in adult fruit flies as suggested by studies in vertebrate cell cultured (White, 2002; Matsuo et al., 2010; Nagata et al., 2012; Tajan et al., 2014) with a positive effect in gut in our conditions (Figures 2D–F). Of note, LOF csw showed no effect on ERK activity in adult fruit flies in various feeding conditions (Supplementary Figure S2).

### Loss-of-Function *csw* Increased Life Span in Poor and Rich Yeast Diets

Our previous observations suggested that LOF *csw* alleles promote life span by reducing insulin signaling. To further understand the relation of CSW with the IIS pathway, we examined whether LOF *csw* affected life span in poor and rich yeast diets as previously reported for mutants of the IIS pathway (Min et al., 2008). In parallel experiments, we analyzed the life span of the IIS mutants *InR^E19^/*+ and *chico^1^/*+ to these diets. As expected, LOF *csw*, *InR*, and *chico* mutants showed extended life span with a normal yeast diet as compared with the control group (Figure 2G). Survival curves for poor diet containing 0.25% of yeast appear to show that all genotypes had a reduced survival compared with themselves on regular fly media (compare Figures 2G,H). Although insulin mutants *InR^E19^/*+ and *chico^1^/*+ showed a higher survival than did the control group, it was smaller compared with that of the LOF *csw^6^/*+ (Figure 2H). Similarly, with a rich diet containing 15% yeast, the insulin mutants *InR^E19^/*+ and *chico^1^/*+ and the LOF *csw^6^/*+ showed a longer life span in rich diet than did the control group (Figure 2I). Of note, median life span of LOF *csw* was insensitive to rich diet (Supplementary Figure S4).

Taken together, these experiments showed that LOF *csw* as well as LOF insulin mutants provided extended life span in poor and rich diets, further supporting a role of CSW in the IIS pathway.

### Suppression of *csw* in Fat Body or Neurons Is Required for Life Span Extension

The effect of LOF *csw* by using the hypomorphic allele *csw*^6^ or *csw*^lf^ was effective in extending life span. The IIS pathway is a key regulator of energy resources in the fat body, neurons, and muscle tissue (Colombani et al., 2003; Géminard et al., 2009; Rajan and Perrimon, 2012; Wang et al., 2014). Thus, we wanted to determine whether LOF *csw* extended life span by acting in those tissues. First, using the GAL4/UAS system (Brand and Perrimon, 1993), we examined the effect of the RNA interference (RNAi) *UAS-csw-RNAi*, which has no predicted off-target sequences and specifically reduced CSW protein levels (Dietzl et al., 2007; Pagani et al., 2009). This *csw-RNAi* produced lethality when it was expressed in the whole body—by *Actin*-*GAL4* or *Tubulin-GAL4*—and a smaller wing phenotype when expressed in the wing by *Bx-GAL4*, similar to the suppression of *RasD85* (Supplementary Figure S5) (Terriente-Félix and de Celis, 2009; Hahn et al., 2013). We drove the expression of *csw-RNAi* to neurons, muscle, and fat body by using previously characterized tissue-specific *GAL4* drivers (*elav^C155^-GAL4*, *DJ757-GAL4*, and *FBr4-GAL4*, respectively) (Lin and Goodman, 1994; Seroude et al., 2002; Lee and Park, 2004; Vrailas-Mortimer et al., 2011). Note that *DJ757-GAL4* also showed some expression in the brain and fat body (Seroude et al., 2002). We confirmed the appropriate expression pattern of those GAL4 drivers by expressing red fluorescent protein (*UAS*-*RFP*) and detected their expression pattern.

**FIGURE 3:**
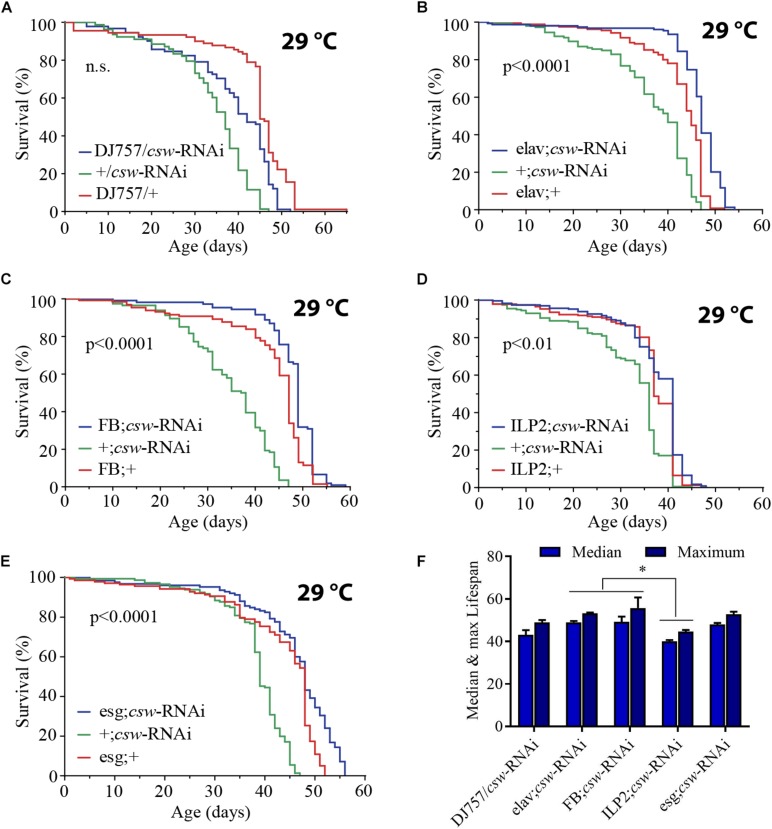
Loss-of-function (LOF) *csw* extends life span acting in specific tissues. **(A–E)** Survival curves showing the effect of the *csw-RNAi* driven to the following tissues: **(A)** Muscle by the specific GAL4 driver (*DJ757/csw-RNAi*) and parental lines (+*/csw-RNAi* and *DJ757/*+). **(B)** Neurons by the specific GAL4 driver (*elav;csw-RNAi*) and parental lines (+*;csw-RNAi* and *elav;*+). **(C)** Fat body by a specific GAL4 driver (*FB;csw-RNAi*) and parental lines (+*;csw-RNAi* and *FB;*+). **(D)** Insulin-producing cells by a specific GAL4 driver (*ILP2;csw-RNAi*) and parental lines (+*;csw-RNAi* and *ILP2;*+). **(E)** Intestinal stem cells and enteroblasts by a specific GAL4 driver (*esg;csw-RNAi*) and parental lines (+*;csw-RNAi* and *esg;*+). Note that *csw-RNAi* increased survival when expressed in neurons, fat body, and gut compared with parental lines. **(F)** Median and maximum life span was higher in flies expressing *csw-RNAi* in fat body or neurons than in IPCs. Statistical significance by log-rank test, corrected for multiple comparisons. The number of animals was *n* = 80 to 149, at least from four independent experiments. ^∗^*p* < 0.05.

Reducing CSW activity in muscle had no effect on survival (Figure 3A). However, expressing *csw-RNAi* in neurons or in the fat body increased life span compared with that in parental controls (Figures 3B,C). These observations are consistent with the current model in which the fat body and neurons control the activity levels of the IIS pathway of all peripheral tissues (Rajan and Perrimon, 2013; Wang et al., 2014). In this model, the fat body senses the feeding state and signals to insulin-producing cells (IPCs) in the brain, which, in turn, promote insulin-like peptide 2 (Dilp2) release. Dilp2 released from IPCs binds to InR in different tissues and activates the IIS pathway (Wang et al., 2014). Because suppression of CSW in neurons extended life span, we wanted to determine whether those neurons were IPCs. Thus, we examined whether *csw-RNAi* expressed in an IPC pattern (*ILP2-GAL4*) modulates life span. Although the statistical analysis indicated a significant difference (*p* < 0.01) (Figure 3D), this manipulation did not affect maximum or median life span. Therefore, other neurons in addition to IPCs must be involved in the *csw*-dependent life span extension (compare Figures 3B,D).

Moreover, the gut appears to be an important determinant of survival in an IIS-dependent manner through systemic and local effects (Biteau et al., 2011; Rera et al., 2012). Thus, we examined life span after suppressing *csw* in intestinal stem cells (ISCs) and enteroblasts by using the specific drivers *esg-GAL4* and *csw-RNAi* (Micchelli and Perrimon, 2006). Expression of *csw-RNAi* increased survival compared with that of the parental lines (Figure 3E).

Of note, whereas suppressing CSW function in neurons or the fat body increased median life span but not maximum life span (Figures 3B,C), the *esg-GAL4;csw-RNAi* increased maximum life span but not median life span. This result of survival (Figure 3), together with the effect of LOF *csw* on AKT activity (Figures 2D–F), might suggest that CSW increased maximum life span through the IIS pathway by local effects, whereas it increased median life span by systemic ones. Furthermore, these data are consistent with a model in which CSW has differential impact on distinct body organs, and those may contribute additively to survival.

These observations showed that by reducing the activity of CSW, life span was extended through action in specific tissues. Consistent with a function of CSW in IIS as a positive regulator, LOF *csw* appeared to extend survival through organs involved in the regulation of energy resources acquired by feeding, like the gut, fat body, neurons, and IPCs (Colombani et al., 2003; Géminard et al., 2009; Rajan and Perrimon, 2012).

### Loss-of-Function *csw* Enhanced Resistance to Starvation With Higher Triglyceride Levels

The studies presented above indicated that CSW controls metabolism and life span through the IIS pathway. Previous reports showed that LOF insulin mutants contain increased lipid content and normal protein levels (Böhni et al., 1999). Fat tissue reservoirs play a key role as energy resource in food shortages, in a way that animals with higher initial adiposity survive longer in starvation (Cherel et al., 1992; Caloin, 2004). Therefore, we wanted to determine whether LOF *csw* mutants showed resistance to starvation and altered lipid and protein content.

**FIGURE 4:**
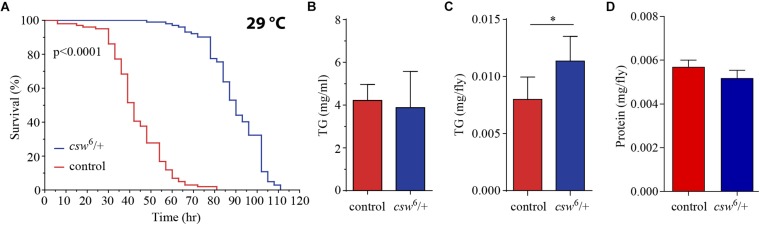
Loss-of-function (LOF) *csw* provides resistance to starvation with increased triglycerides levels. **(A)** Survival curves showing higher resistance to starvation in LOF *csw* mutant compared with control group, log-rank test (*n* = 101 and 102, respectively, from five independent experiments). **(B,C)** Triglyceride levels of LOF *csw* mutant compared with control group, in hemolymph (mg/ml) **(B)** (*n* = at least four independent experiments); bars, mean ± SEM (*p* > 0.05 *t*-test) and in abdominal segment **(C)** (*n* = 10) (^∗^*p* < 0.05 Mann–Whitney test); bars, median ± interquartile range (IQR). **(D)** Protein levels of LOF *csw* mutant compared with control group, *n* = 10, *p* > 0.05 *t*-test.

First, we examined starvation resistance in adult LOF *csw* fruit flies maintained in a fly media composed of agar 1%, which provide mainly a source of water. We found that LOF *csw* flies had high resistance to starvation as compared with the control group (Figure 4A). Next, we examined circulating TG content in hemolymph, as well as total content of TG in the abdominal segment, where most of the fat body is located. LOF *csw* showed normal levels of TG in hemolymph but increased levels in the abdominal segment as compared with control group (Figures 4B,C), whereas protein levels were unaffected (Figure 4D). The higher resistance to starvation can be explained by the higher TG content when starvation began (Caloin, 2004).

Taken together, these results are consistent with a role of CSW in IIS signaling mechanisms (Figure 2). Moreover, the extended life span of LOF *csw* in starvation and rich yeast diet (Figures 2, 4) strongly suggests a role of CSW in metabolic regulation.

### Loss-of-Function *csw* Impairs Metabolic Rate Homeostasis

To gain insight into how the PTP CSW is related to metabolism, we measured the metabolic rate at rest and during activity in single fruit fly experiments using a high-resolution setup at constant temperature (25°C). Young (3–5 days) and old (37–39 days) LOF *csw* or control females were transferred to the respirometry chamber, and the CO_2_ production together with the levels of activity and temperature was recorded in real time.

**FIGURE 5:**
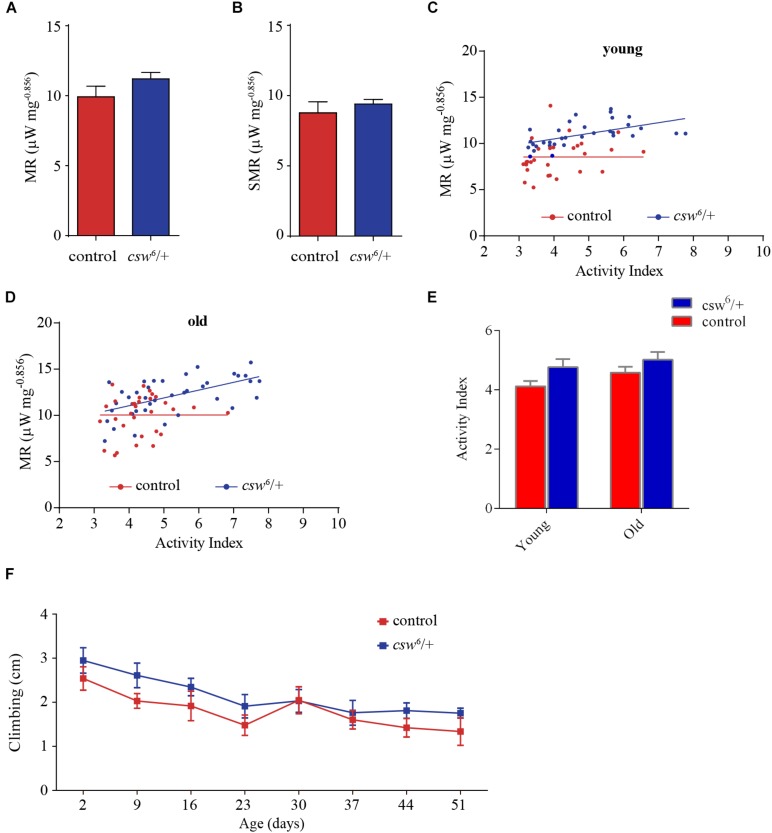
Loss-of-function (LOF) *csw* impairs metabolic rate homeostasis. **(A,B)** Mass-corrected metabolic rate (μW mg^– 0.856^) **(A)** and mass-corrected standard metabolic rate (μW mg^– 0.856^) **(B)** in LOF *csw* mutants (*n* = 10) compared with control group (*n* = 9). Bars, means ± SEM. **(C,D)** Linear regression of metabolic rate (MR) over activity index in young (3–5 days) **(C)** and old fruit flies (37–39 days) **(D)** in LOF *csw* and control group (*n* = 28–35). Each point represents a 5-min trace recording. Note that LOF *csw* flies show a significant linear relation of MR over activity index (*p* = 0.0008) and (*p* = 0.0001) for young and old flies, respectively. **(E,F)** Activity levels measured in experiments of respirometry within age group **(E)** (*n* = 28–35), and climbing assays throughout life **(F)** were not affected by LOF *csw* compared with control group, two-way ANOVA followed by Sidak’s test *p* > 0.05. For panel **(F)**, the number of animals was *n* = 211 to 234 from 10 independent experiments. **(E,F)** Mean ± SEM.

Mass-specific metabolic rate and mass-specific standard or resting metabolic rate of LOF *csw* animals were indistinguishable from those of the control group in young (Figures 5A,B) and old fruit flies (Supplementary Figure S6). Consistent with studies on IIS pathway mutants (Hulbert et al., 2004), LOF *csw* produced no effect on average metabolic rate.

Interestingly, in a regression analysis of the average metabolic rate over activity index, there was no significant slope in the control group (i.e., young and old flies), showing that the metabolic rate appears to be constant at different levels of activity (Figures 5C,D). In contrast, LOF *csw* appears to lack this homeostatic mechanism because the metabolic rate increased together with their activity levels, showing a significant slope in young (*p* = 0.0008, *R*^2^ = 0.31) and old animals (*p* = 0.0001, *R*^2^ = 0.33) (Figures 5C,D). In other words, CSW seems to contribute in the maintenance of a constant metabolic rate with larger activity because such functional property was lost in *csw* mutants.

Although there was a main effect of genotype over activity, the LOF *csw* fruit flies in each age group showed normal average activity levels as compared with the control group in respirometry assays (Figure 5E) and in climbing assays throughout life (Figure 5F).

This result seems to provide evidence of the existence of a homeostatic mechanism for metabolic rate, in which CSW and perhaps the IIS pathway might be involved.

### Overexpression of Gain-of-Function *csw* in the Fat Body Reduced Life Span

Our experiments indicated that CSW may have a role in normal physiology affecting the IIS pathway, metabolic rate, and life span. Therefore, it is possible that similar alterations would be implicated in cases of NS with mutated SHP2. However, this and related disorders are largely produced by GOF mutations in components of the RAS-ERK1/2 signaling pathway, which produce an enhancement of this pathway. Thus, to test the potential clinical relevance of the *csw*-dependent effects on life span, we analyzed the effect of enhancing CSW functions by overexpressing a WT allele and a GOF one, which show the same expression level of CSW protein (Pagani et al., 2009), allowing us to exclude differences from these transgenes owing to expression levels.

**FIGURE 6:**
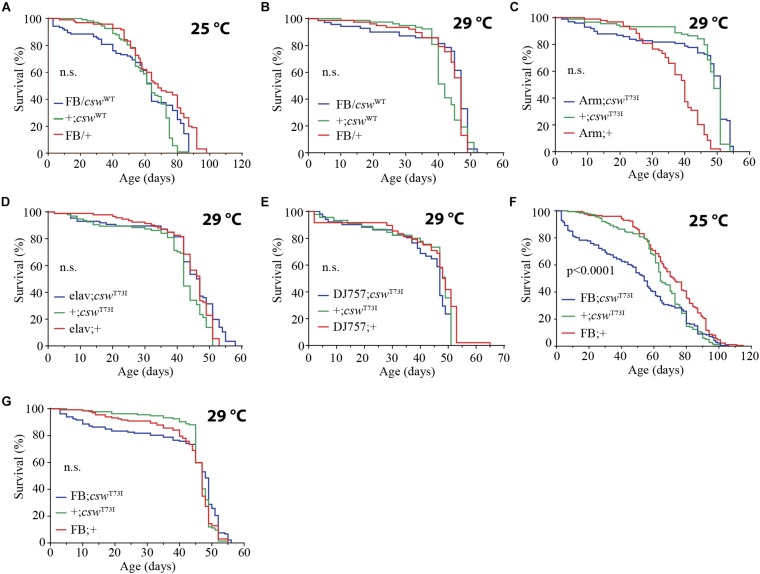
Gain-of-function (GOF) *csw* T73I allele in fat body, but not wild-type (WT) *csw* allele, reduced life span. **(A,B)** Effect of overexpressing WT *csw* in fat body at 25°C **(A)** and at 29°C **(B)**. Note survival early in life at 25°C of the *FB/cswWT* compared with parental lines (*FB/*+ and *cswWT/*+) (*p* > 0.05). **(C–G)** Effect of GOF *csw-T73I* transgene targeted to specific tissues on life span. **(C)** Survival curves after overexpressing GOF *csw-T73I* to all cells of fruit flies by a weak driver (*Armadillo-GAL4*) compared with parental genotypes (*p* > 0.05). **(D)** Survival curves showing the effect of overexpressing GOF *csw-T73I* driven to neurons by a specific GAL4 driver (*elav;csw-T73I*) and parental lines (+*;csw-T73I* and *elav;*+) (*p* > 0.05). **(E)** Overexpressing GOF *csw-T73I* in muscle by a specific GAL4 driver (*DJ757;csw-T73I*) compared with parental lines *(*+*;csw-T73I and DJ757;*+) (*p* > 0.05). **(F,G)** Survival curves of flies overexpressing GOF *csw-T73I* in fat body by a specific GAL4 driver (*FB; csw-T73I*) and parental lines (+*;csw-T73I* and *FB;*+) in experiments performed at 25°C **(F)** or at 29°C **(G)**. Note that GOF *csw-T73I* significantly reduced survival at 25°C, whereas at 29°C, a non-significant tendency in early life up to 40 days old is shown. Log-rank test, corrected for multiple comparisons for all panels and Wilcoxon test in panel **(A,F,G)**. Number of animals was *n* = 86 to 134 at least from four independent experiments.

Ubiquitous overexpression of *csw* WT with the *Armadillo-*GAL4 driver (*Arm-GAL4;csw^WT^*) or in specific tissues such as neurons, muscle, or fat body using the same GAL4 drivers used for *csw-RNAi* expression (i.e., *elav-GAL4*, *DJ757-GAL4*, and *FBr4-GAL4*) did not alter life span (Figures 6A,B and Supplementary Figure S7). This indicated that an increase in CSW function of a WT protein has no specific or non-specific deleterious effects.

Next, we examined the effect on life span of the GOF *csw* allele T73I, which models the most frequent human mutation involved in leukemia in NS (Oishi et al., 2006; Chan and Feng, 2007) and express fivefold more CSW protein than did the non-transgenic control flies (Pagani et al., 2009). As expected, the expression of the GOF *csw* allele T73I (*UAS-csw-T73I*) to the wing by using *Bx-GAL4* produced an ectopic wing vein (Supplementary Figure S8), confirming its GOF effect in development as previously reported (Oishi et al., 2006). The expression of the GOF *csw* allele T73I (*UAS-csw-T73I*) in the whole body by *Tub-GAL4* was lethal, whereas the *armadillo-*GAL4 driver (*Arm-GAL4/csw^T73I^*) produced viable progeny. However, the overexpression of the T73I allele in the whole body or in specific tissues such as neurons or muscle did not cause any effect on life span (Figures 6C–E). In contrast, overexpressing the GOF *csw* allele T73I in the fat body reduced life span as compared with control groups at 25°C (Figure 6F) but not at 29°C (Figure 6G), although both curves showed a similar tendency in early life. Consistent with that finding, the overexpression of the *csw* WT allele in the fat body produced a similar, but not significant, decrease in survival in the first portion of the life span curve when flies were cultured at 25°C but had no effect when cultured at 29°C (Figures 6A,B). Most importantly, these data also indicate a specific effect of the GOF *csw* mutation, but not an effect of overexpression, because overexpressing WT *csw* transgene did not affect life span (Figures 6A,B).

Altogether, these results further support a role of CSW in the regulation of fat body functions related with the IIS pathway. Surprisingly, it was easier to extend life span than reduce it by manipulating *csw*, because only the manipulations in fat body could reduce life span (Figures 1, 3, and 6). However, this fact strengthens the idea of a specific role of *csw* in the fat body, instead of a non-specific effect of transgenic suppression of *csw*. Therefore, these data predict that GOF SHP2 may have a pathological effect in humans and expand the current model of molecular pathogenesis in NS of enhanced RAS-ERK1/2 signaling (see *Discussion*).

## Discussion

These studies advanced our understanding on the role and mechanisms of the phosphatase CSW/SHP2 in adult organisms, which appears to be unrelated with the regulation of ERK1/2 signaling activity, previously described in a context of development, as well as long-term memory deficits in adult animals (Jindal et al., 2015). The study also provides insight into insulin-mediated processes and their underlying mechanisms, which is of great interest for human health owing to its essential roles in diabetes, metabolism, aging, and cancer (Reyes-DelaTorre et al., 2012; Rajan and Perrimon, 2013). Overall, this study showed that CSW plays an important role in life span in a signaling pathway involving InR and chico.

Taken as a whole, these findings expand the current model of the molecular pathogenesis in NS (i.e., enhanced RAS-ERK1/2 signaling) and predict a more comprehensive model that includes altered RAS-ERK1/2 and insulin signaling mechanisms as well, affecting metabolism throughout life. Importantly, we showed that CSW has distinct effects on molecular signaling in development and in adult animals. This fact has important implications on the molecular pathogenesis of NS and related disorders. For instance, the rational drug design for NS and related disorders has been directed to reducing the enhanced RAS-ERK1/2 signaling (Li et al., 2005; Wang et al., 2012), which might be not appropriate for adult subjects or metabolic alterations. The clinically relevant GOF CSW mutant protein acting in the fat body reduced survival (Figure 6), predicting altered metabolism throughout life in subjects carrying GOF SHP2 mutant proteins. This prediction is consistent with the resistance to diet-induced obesity observed in a mouse model of NSML and its correlation in patients (Tajan et al., 2014).

### CSW and Life Span

We showed that the phosphatase CSW participates in the regulation of life span, similar to other classical insulin pathway mutants in several features. The *csw*-dependent life span effect was positively and negatively modulated by LOF and GOF alleles in the fat body, respectively. However, a functional role of CSW in other important tissues for nutrient-dependent metabolism (IPCs, neurons, and gut) was revealed by a *csw-RNAi*. Because overexpressing *csw* WT allele did not affect survival (Figures 6A,B and Supplementary Figure S7), we can exclude a gene–dose effect of the GOF allele. This supports the notion that the effect of the GOF allele is specific to the mutant allele, as previously observed for several GOF alleles in a context of memory (Pagani et al., 2009). However, even when CSW plays a role in different organs or tissues (Figure 3), GOF *csw* only produced a significant effect on life span when it was expressed in the fat body (Figure 6). This defines a clear difference between CSW normal functions and GOF CSW effects. Hence, it is possible that the LOF *csw* allele, in contrast to the GOF allele, produced a genetic dosage-dependent effect. Alternatively, the LOF *csw* may be acting as the NSML-associated alleles, which produce some phenotype owing to the loss of the PTP catalytic action, whereas other phenotypes, such as the loss of an anti-apoptotic effect, depended on the C-SH2 domain, which is PTP and ERK1/2 independent (Stewart et al., 2010).

In addition, we did not detect an effect of LOF CSW at the levels of pERK or pAKT compared with the control genotype (Supplementary Figure S2), whereas reduced pAKT levels were detected at the posterior midgut, and no pERK was detected in the same gut region (Figures 2D–F and Supplementary Figure S3). These observations suggested that the average amount of pAKT and pERK was similar in LOF CSW and control genotype, at least when a mix of all tissues of the body was analyzed. This might be possible if the intensity and the time course of signaling activity would be dissimilar in different tissues, and some differences in a specific tissue might be diluted on the average. A dissimilar activity can be expected because the CSW expression levels differ substantially in different tissues (Graveley et al., 2011). Also, AKT but not ERK was activated by refeeding in the gut, at least after 16 h of fasting followed by 4 h of refeeding (Figures 2D–F). In the same line of thought, when we examined pAKT by immunostaining, we found a clear pAKT staining with 4 h of refeeding in the posterior midgut, but not in more anterior regions of the gut. Therefore, these data suggested that the activity of AKT and ERK after refeeding will take place at different times in different organs of the body and even within the same organ.

More important, regardless of the levels of pAKT, the LOF *csw* allele impaired the localization of pAKT in plasma membrane and in the nucleus (Figure 2F), which might be even more significant to disrupt an effective signal transduction than the total levels of activated AKT. Consequently, it is possible to expect that some effects of the manipulations of csw might depend not necessarily on the levels of phospho-proteins but on the impaired sub-cellular localization or in the time course of signaling transduction, as we previously showed for pERK in the fly head after olfactory conditioning (Pagani et al., 2009).

### CSW and the Insulin-Like Growth Factor Signaling Pathway

**FIGURE 7:**
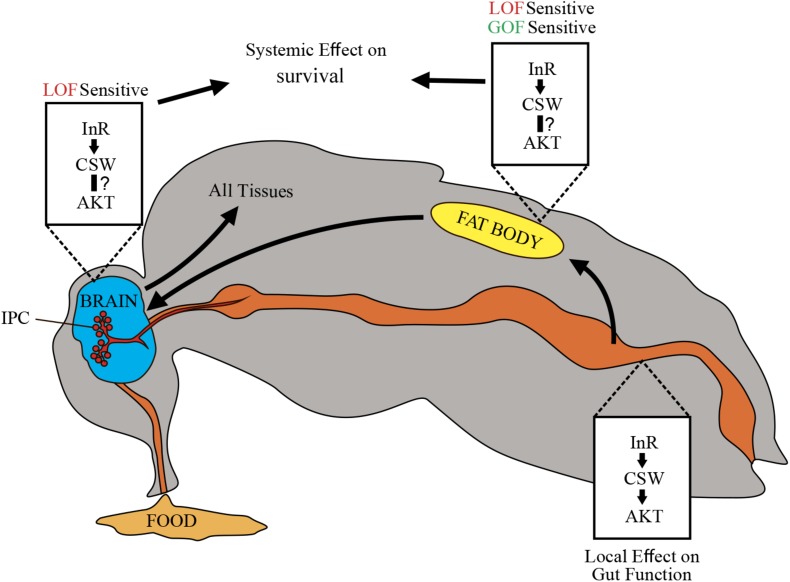
Schematic representation of potential CSW-dependent mechanisms for life span regulation. CSW-mediated life span modulation depending on organs regulating energy resources acquired by feeding, like gut, fat body, and neurons. Arrows between them indicate subsequent effects. Through the insulin-like growth factor signaling (IIS) pathway, CSW in fat body and neurons produced systemic effect on all peripheral tissues such as metabolic homeostasis and aging delay. Likely, CSW acting in gut mediates IIS pathway-dependent local effects (see Figure 3E). Different tissues are not equally sensitive to loss-of-function (LOF) and gain-of-function (GOF) *csw* alleles.

It was previously established that SHP2 participates as a molecular component downstream of InRs; however, its role on metabolism and survival was obscure. In this study, we identified that the PTP CSW controls life span through modulation of the IIS pathway (Figures 1, 2) (Tatar et al., 2001). This effect appeared to be mediated by systemic positive and negative regulatory roles on survival by acting in the fat body, which in turn may affect IPCs and the Dilp2-dependent peripheral tissues (Figures 3–6) (Géminard et al., 2009; Rajan and Perrimon, 2012). This interpretation supports a key role of the IIS pathway in fat body, as well as a systemic effect of fat body, which is consistent with the current understanding of fat body in the regulation of metabolism (Colombani et al., 2003; Géminard et al., 2009; Rajan and Perrimon, 2012; Wang et al., 2014). Comparably, transgenic mice expressing a dominant-negative form of SHP2 showed insulin resistance in muscle and adipocytes (Maegawa et al., 1999), whereas tissue-specific reduction of SHP2 function in hepatocytes or adipocytes showed increased tolerance to insulin and high-fat diets (Matsuo et al., 2010; Nagata et al., 2012). Consistently, systemic and local insulin-dependent modulation was reported (Wang et al., 2014). In this regard, whereas suppressing CSW function in neurons or the fat body increased median life span, it did not affect maximum life span (Figures 3A–C). However, suppressing CSW function in the gut increased maximum life span but not median life span (Figure 3E). This suggested that *csw* operates differently in the gut (e.g., by a local effect) whereas, in the fat body or neurons, it is expected to regulate life span through a systemic effect (Figure 7).

We did not provide direct evidence that the reduced life span in CSW GOF was mediated by IIS pathway. Yet a number of observations provide a clear possibility. First, csw LOF prolongs life span by interacting with *InR^E19^* and *chico*^1^ and impairs the activation and localization of pAKT, suggesting that IIS is involved in the effects of CSW on life span. Additionally, CSW prolonged life span in poor and rich diets and increased TG levels like insulin signaling mutants. Finally, when we examined which tissues might be involved in CSW LOF effects, we found that csw-RNAi in fat body reduced life span, supporting the idea that IIS is involved in the effects of CSW on life span. Altogether, these data indicated that IIS might be involved in the effect of CSW GOF on life span.

However, we found that by overexpressing the GOF *csw* allele T73I in fat body, life span was strongly reduced at 25°C, whereas at 29°C, only a small but not significant tendency was detected in early life (Figures 6F,G). Consistently, a similar tendency was observed in fruit flies overexpressing WT CSW protein in fat body at 25°C compared with 29°C (Figures 6A,B). Therefore, in GOF and WT transgenic flies, a reduced survival in early life appeared more evident at 25°C. This result did not depend on a thermosensitive GOF or WT CSW proteins with reduced activity at 29°C because these proteins are functional at 30°C, producing a specific effect in long-term memory (Pagani et al., 2009), as well as at 29°C, producing lethality in the case of GOF protein expressed in the whole body (see section “Overexpression of Gain-of-Function *csw* in the Fat Body Reduced Life Span”).

One possible explanation for the effect of GOF CSW at 25°C, but not at 29°C, is that this protein in fat body shows a genotype × environment interaction. A more complex possibility is that the dynamics of the GOF CSW protein at 29°C becomes functionally more like its interacting proteins (i.e., CHICO, PI3K, and RAS) (Figure 2A). The dynamics of activation of the GOF CSW protein is faster than the WT protein (Keilhack et al., 2005), but GOF and WT transgenes produced a faster signaling activation than the endogenous CSW protein (Pagani et al., 2009). Moreover, the molecular dynamics of WT proteins is expected to be faster at 29°C than at 25°C (i.e., Q10 of 2–3 for most enzymes). Thus, in a condition where the molecular dynamics of all cells is speeded up by temperature at 29°C, the enhanced activity of GOF CSW protein may become more compatible to the molecular context, reducing the severity of the life span impairment. This idea implies that the activity of the GOF CSW protein is proportionally less affected by temperature than its interacting WT proteins. Such condition perhaps can be achieved because T73I GOF CSW has shifted the basal equilibrium state toward the active state (i.e., ∼10-fold higher basal activity than the WT CSW protein) but appear to be ∼4-fold less responsive to stimulation than the WT CSW protein because it is almost at the top activity in the basal state (Keilhack et al., 2005).

### CSW and Metabolic Rate Homeostasis During Activity

We found that the control genotype showed a constant average metabolic rate at different levels of activity (Figures 5C,D). In contrast, LOF *csw* mutants showed a significant regression of metabolic rate over activity index (Figures 5C,D). This observation suggested that CSW contributes to the maintenance of a constant metabolism because it was lost with LOF CSW. Because we performed these experiments in fruit flies fed *ad libitum*, it is expected that they consume mainly carbohydrates, and, therefore, the relation between CO_2_ production and O_2_ utilization, or RQ, would be equal to 1 in both genotypes (Wang et al., 2008). Therefore, a larger CO_2_ production cannot be explained by an altered RQ; even if the LOF *csw* flies use more fat or proteins as their energy resource, RQ will be reduced by increased oxygen consumption (Wang et al., 2008). However, an RQ above 1 has been reported in patients with overfeeding, which may explain an enhanced CO_2_ production (Hulst et al., 2005). Levels of energy consumption during activity can vastly exceed the levels required at rest. For instance, *Drosophila* in flight require up to 70-fold more oxygen than at rest (Wegener, 1996). However, this cannot explain the observed differences because there was no change in activity within age group in respirometry or in climbing assays throughout life (Figure 5), suggesting that other mechanisms might be involved. In fact, it was reported that the GOF *csw* allele E76K increases oxygen consumption, mitochondrial respiratory function, and reactive oxygen species (ROS) generation (Zheng et al., 2013). Although CO_2_ production was not analyzed in that report, it is feasible that the LOF *csw* allele also affects mitochondrial function underlying the effect reported here (Figures 5C,D). In fact, oxygen consumption rate is higher in mitochondria of SHP2 knock-out liver cells (Nagata et al., 2012), although the reason for our observation on metabolic rate remains unclear, strongly indicating a loss of metabolic homeostasis in the LOF *csw* fruit flies, supporting its role in metabolism.

Taken together, our experiments showed that manipulations of CSW in *Drosophila* can be used to study the relationship between organs in metabolism as well as its role in NS throughout adult life. This establishes a solid base to study in detail the effect of mutations modeling NS and NSML in fruit flies, which will allow us to learn about these disorders and insulin functions as well.

## Data Availability Statement

All datasets generated for this study are included in the article/Supplementary Material.

## Author Contributions

LR, PS, AS, BG, and MP contributed to the conception and design of the study. LR, PS, AS, SL, and MP designed the experiments and collected and interpreted the data. SL designed the equipment and software. LR, PS, SL, and MP performed the statistical analysis. LR, PS, BG, and MP wrote the first draft of the manuscript. All authors contributed to manuscript revision and read and approved the submitted version.

## Conflict of Interest

The authors declare that the research was conducted in the absence of any commercial or financial relationships that could be construed as a potential conflict of interest.
