# Supplementary material for: The Phosphatase CSW Controls Life Span by Insulin Signaling and Metabolism Throughout Adult Life in Drosophila
Source: Front Genet. 2020 May 7;11:364. doi: 10.3389/fgene.2020.00364 (PMC7221067; doi:10.3389/fgene.2020.00364)
Supplement: Supplementary file 1 [file Data_Sheet_1.pdf]

Supplementary Material

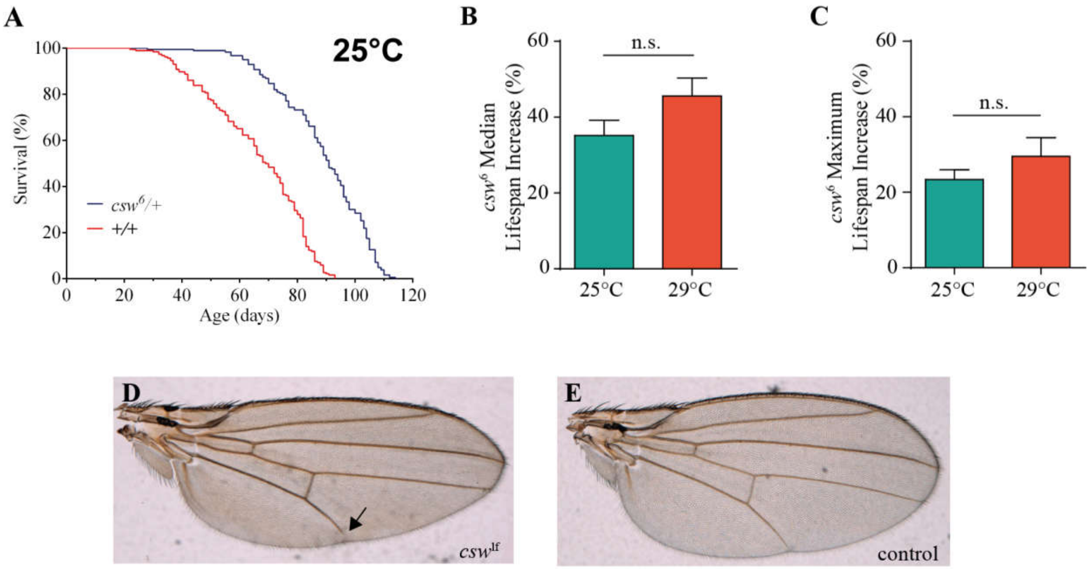

Supplementary Figure 1. LOF *csw* phenotypes in lifespan and wing veins. (A) Effect of the LOF *csw*^{6} allele on lifespan at 25°C, p<0.0001, Log-Rank test. The number of animals was n= 210 to 227*,* from nine independent experiments. **(B-C)** Proportion of the effect of the LOF *csw*^{6} allele on median **(B)** and maximum lifespan **(C)** at 25°C (n=9) and 29°C (n=4); p>0.05, t-test. **(D)** LOF phenotypes as incomplete L5 vein (arrow) produce by the *csw*^{lf} allele compared with control *w*^{1118}**(E)**.

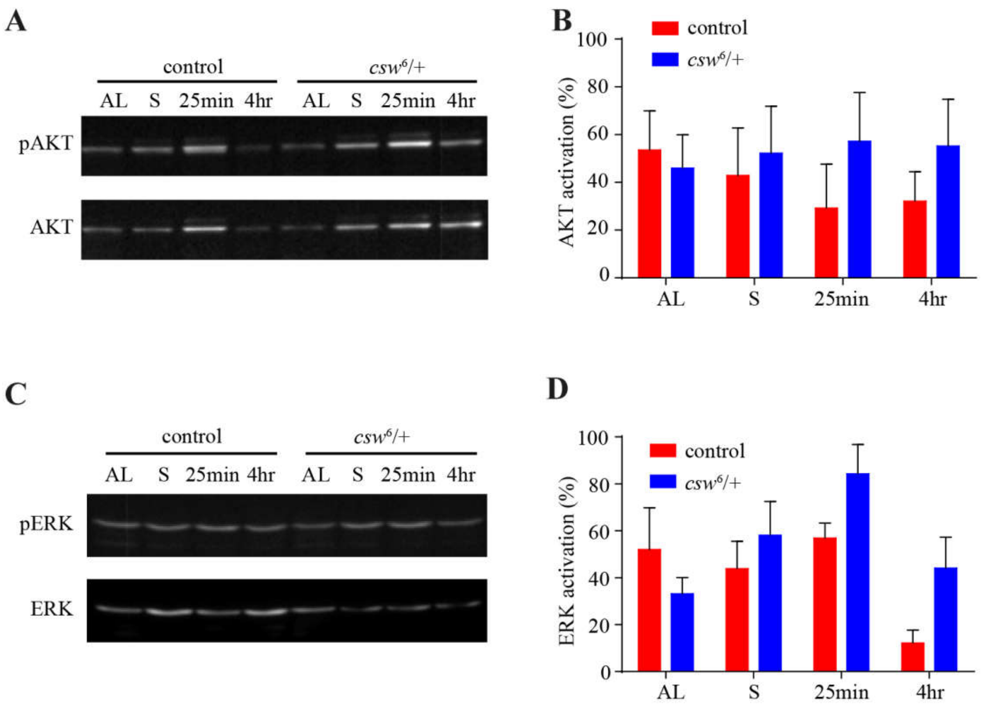

Supplementary Figure 2. (A-D) Western blot of adult fly extracts in control and *csw* mutant flies using anti-AKT and anti-phospho-AKT, showing blot example **(A)** and AKT activation as pAKT/AKT **(B);** andusing anti-ERK and anti-phospho-ERK, showing blot example **(C)** and ERK activation as pERK/ERK **(D)**. Feeding treatments were, ad libitum (AL), starved for 16 hr (S), 25 min after refeeding (25min) and 4 hr after refeeding (4hr). n=4 in all cases, except for AKT and pAKT 25min n=3. Two-way ANOVA, $p>0.05$.

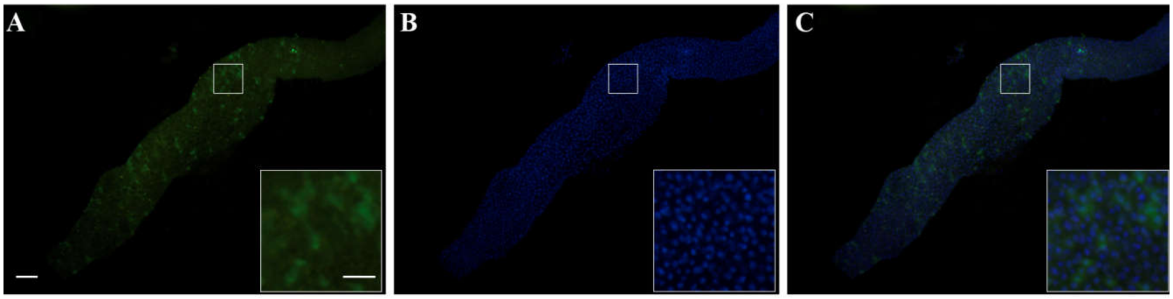

Supplementary Figure 3. Activation of ERK and AKT in adult tissues. Immunostaining in young midgut of control flies after 1% H_2O_2 for 16 hr showing **(A)** dp-ERK staining, **(B)** DNA staining using DAPI and **(C)** Merge. Bar represents 100 μm, inset 25 μm.

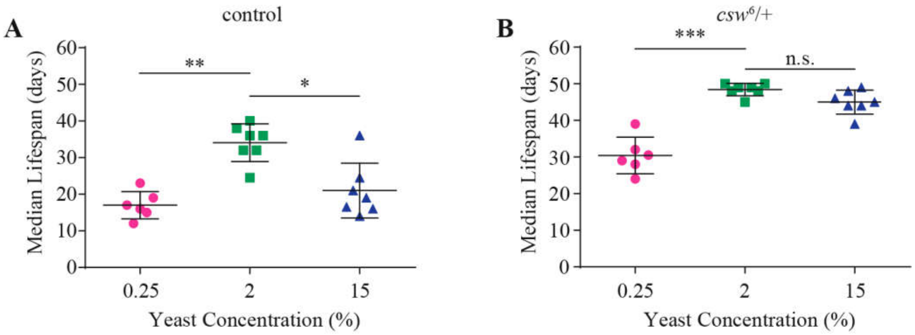

Supplementary Figure 4. Effect of rich and poor yeast diet on survival. Median lifespan recorded in control **(A)** and LOF *csw*(**B)** flies cultured in different yeast concentrations. Each data point represents an independent experiment including 25-30 animals per data point (Median ± standard deviation). Note that LOF csw^6 was insensitive to high yeast concentration (15%). Asterisks indicate * $p<0.05$, ** $p<0.01$ and *** $p< 0.001$, ANOVA Kruskal-Wallis.

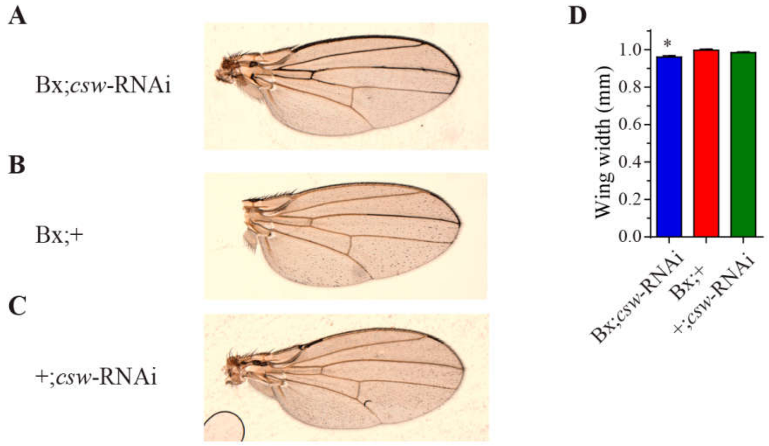

Supplementary Figure 5. Wing phenotype produced by csw-RNAi. (A-C) Effect of *csw-RNAi* expressed in the dorsal wing disc (*Bx;csw-RNAi*) **(A)** and parental controls **(B** and **C)**. **(D)** Quantification of wing width in *Bx;csw-RNAi* compared with parental controls Bx;+ ($p<0.01$) and +;csw-RNAi ($p<0.05$), n=8, ANOVA followed by Dunnett test.

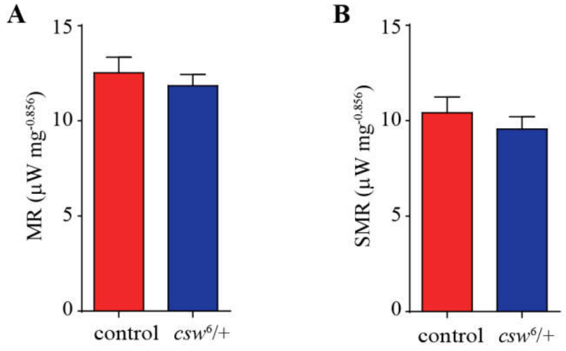

Supplementary Figure 6. Mass specific metabolic rate and standard metabolic rate in old flies. (A) Mass-corrected metabolic rate (µWatts/mg$^{-0.856}$). LOF *csw* mutants (n=10) compared with control group (n=9). **(B)** Mass-corrected standard metabolic rate (µWatts/mg$^{-0.856}$). Old (37-39 days) LOF *csw* mutants (n=8) compared with control group (n=8). Bars, means ± SEM. t-test $p>0.05$.

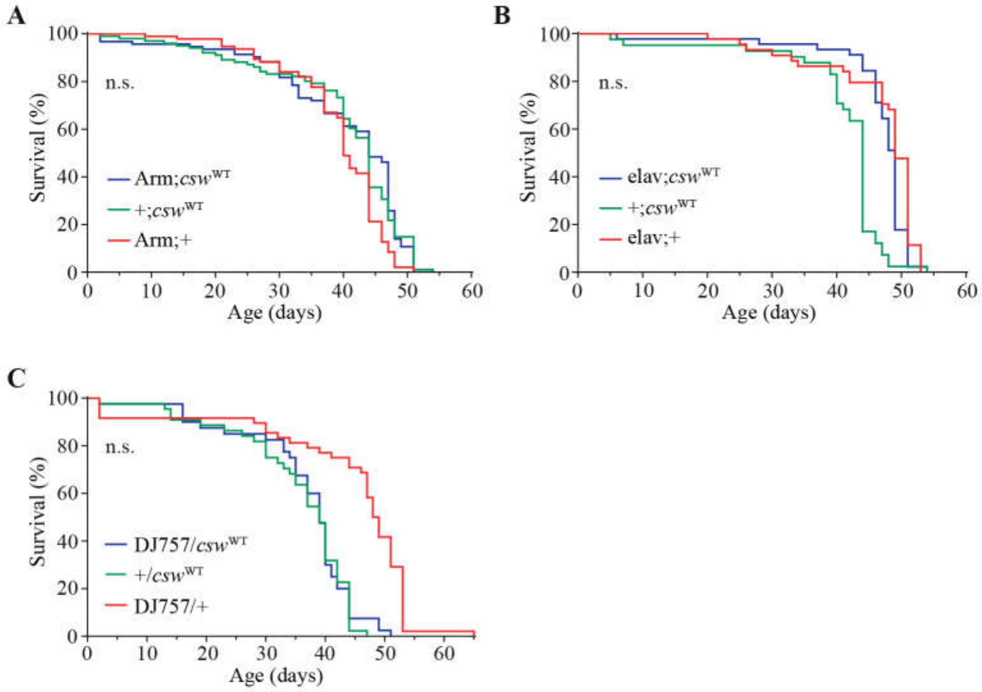

Supplementary Figure 7. Overexpression of csw^{WT} allele in all cells of the body, neurons or muscle did not affect lifespan. (A-C) Survival curves showing no effect of csw^{WT} driven to specific tissues by GAL4. **(A)** All the cells of the body (Arm;csw^{WT}) and parental lines (+;csw^{WT} and *Arm;*+). **(B)** Neurons by the specific GAL4 driver (*elav;*csw^{WT}) and parental lines (+;csw^{WT} and *elav;*+). **(C)** Muscle by the specific GAL4 driver (*DJ757/*csw^{WT}) and parental lines (+/csw^{WT} and *DJ757*/+). Log-Rank test, corrected for multiple comparisons.

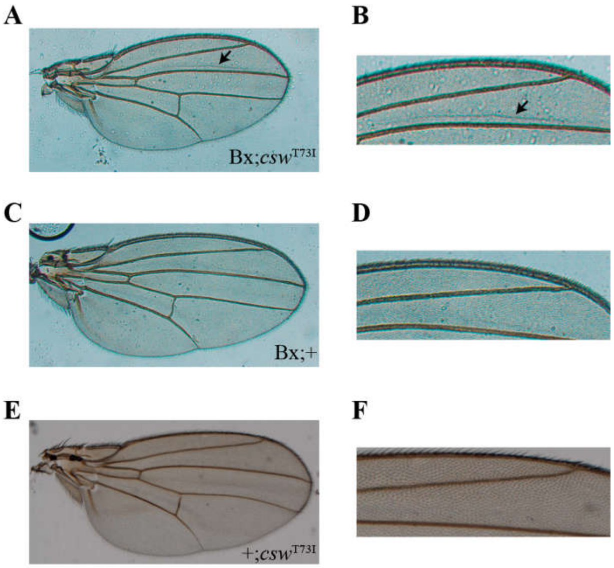

Supplementary Figure 8. Ectopic wing vein produced by GOF *csw*^{T73I} allele. Wing vein phenotype (arrow) produced by GOF *csw*^{T73I} allele expressed in wing by Bx-GAL4 **(A-B)** and wings in parental controls **(C-F)**.
